# Pulmonary Embolism in Antiphospholipid Syndrome (APS)—Where Are We and Where Are We Going?

**DOI:** 10.3390/ijms27020895

**Published:** 2026-01-15

**Authors:** Mateusz Lucki, Bogna Grygiel-Górniak, Ewa Lucka, Maciej Lesiak, Aleksander Araszkiewicz

**Affiliations:** 1 Department and Clinic of Cardiology, University of Medical Sciences, 60-545 Poznań, Poland; mateuszlucki@ump.edu.pl (M.L.); maciej.lesiak@ump.edu.pl (M.L.); aaraszkiewicz@ump.edu.pl (A.A.); 2 Department of Rheumatology, Rehabilitation and Internal Diseases, Poznan University of Medical Sciences, 61-701 Poznań, Poland; bgrygiel@ump.edu.pl; 3 Clinical Rehabilitation Laboratory, Department of Rehabilitation and Physiotherapy, University of Medical Sciences, 60-545 Poznań, Poland

**Keywords:** antiphospholipid syndrome, epidemiology, pulmonary embolism, risk factors, treatment

## Abstract

Pulmonary embolism (PE) is one of the most serious complications of antiphospholipid syndrome (APS), a systemic autoimmune disorder defined by thrombotic events and persistent antiphospholipid antibodies (aPLA). PE occurs in 11–20% of patients and may constitute the initial clinical manifestation. Young and middle-aged women are most frequently affected, and triple-positive aPLA profiles markedly increase the risk of recurrence and long-term morbidity, including chronic thromboembolic pulmonary hypertension (CTEPH). This review article summarizes current evidence on the epidemiology, pathophysiology, diagnostic approach, and management of PE in APS. Key mechanisms include anti-β2-glycoprotein I-mediated endothelial and platelet activation, complement engagement, and neutrophil extracellular trap formation, resulting in immunothrombosis. Diagnostic pathways follow standard PE algorithms; however, chronically elevated D-dimer levels and lupus anticoagulant-related aPTT prolongation require careful interpretation and consideration. Long-term vitamin K antagonist therapy remains the standard of care, whereas direct oral anticoagulants are not recommended in high-risk APS. Future directions include improved risk stratification through detailed aPLA profiling and the use of emerging biomarkers, early screening for CTEPH, and the development of targeted therapies such as complement inhibition and anti-NETosis strategies.

## 1. Introduction

Antiphospholipid syndrome (APS) is a systemic autoimmune disease characterized by clinical symptoms (vascular thrombosis or pregnancy complications) and the presence of antiphospholipid antibodies (aPLA). In clinical practice, three distinct forms of APS are recognized: primary APS (pAPS)—occurring in the absence of other autoimmune diseases, secondary APS,—most commonly associated with systemic lupus erythematosus (SLE), and less frequently with primary Sjögren’s syndrome, rheumatoid arthritis, systemic sclerosis, systemic vasculitis, or dermatomyositis; and catastrophic antiphospholipid syndrome (CAPS)—a rare but severe form characterized by rapidly progressive multiorgan thrombosis and high mortality [1,2]. Systemic complications of APS include thrombotic endocarditis, valvular dysfunction, cerebrovascular obstruction, proliferative nephritis, deep vein thrombosis, and PE. Most of these complications are potentially life-threatening and prompt diagnosis and treatment. The development of APS-related PE is driven by the pathogenic activity of antiphospholipid antibodies, particularly anti-β2-glycoprotein I (anti-β2-GPI) antibodies, which play a central role in initiating thrombotic processes through endothelial activation and dysfunction [3,4,5].

The most common macrovascular APS manifestations include deep vein thrombosis, PE, and stroke. Among them, PE is the most common pulmonary manifestation of APS, occurring in 11% to 20% of individuals. Furthermore, PE may be the first manifestation of APS in approximately 9% to 12% of patients (summarized by Gaspar et al.) [6]. Thrombotic APS is characterized by a wide spectrum of clinical symptoms that are not always easy to recognize, requiring thorough diagnostics and clinical vigilance [7]. Despite appropriate treatment, patients with APS experience recurrent thrombotic events, characterized by symptoms similar to those of the initial episode [8,9,10]. As a result, in retrospective analyses, the incidence of PE increases from 5.2% to approximately 12% over the next 10 years (Euro-Phospholipid cohort) [10].

Considering the increased thrombotic risk in patients with APS-related PE, this review aims to summarize current evidence on the molecular mechanisms, diagnostic strategies, and therapeutic approaches, emphasizing the complexity of the underlying pathophysiological processes and key mechanisms involved in disease progression.

## 2. Review Methodology

This article was designed as a narrative review and prepared in accordance with the SANRA (Scale for the Assessment of Narrative Review Articles) guidelines. The aim of the literature search was to identify and synthesize key publications relevant to PE in antiphospholipid syndrome, rather than to perform a systematic evidence appraisal. A comprehensive but non-systematic search of the literature was conducted using PubMed, MEDLINE, Web of Science, Scopus, and DOAJ, covering publications from January 2000 to November 2025. The search strategy combined MeSH terms and free-text keywords, including: “Antiphospholipid Syndrome,” or “APS,” and “Pulmonary Embolism,” or “PE”.

The selection of publications was guided by their relevance to the topic, scientific quality, and contribution to understanding the epidemiology, pathophysiology, diagnosis, and management of APS-related pulmonary embolism. Priority was given to original clinical studies, cohort and registry data, mechanistic and immunopathological research, narrative and systematic reviews, meta-analyses, and international guidelines (e.g., EULAR, ASH, ESC). Case reports, conference abstracts, editorials, non-indexed sources, and studies without direct clinical applicability were excluded.

Study selection was based on qualitative assessment of titles, abstracts, and full texts by the authors, without predefined quantitative selection thresholds. The final body of literature was used to provide a structured narrative synthesis across three principal domains: epidemiology and clinical presentation, pathophysiological mechanisms, and diagnostic and therapeutic strategies, including long-term outcomes and future perspectives.

## 3. Epidemiology

### 3.1. Epidemiology of APS

Antiphospholipid syndrome (APS) is a relatively rare autoimmune disorder, with reported prevalence ranging between 40 and 50 cases per 100,000 individuals and an estimated annual incidence of approximately 1–5 new cases per 100,000 population [11,12,13,14,15]. Variability in epidemiological estimates across regions reflects differences in study design, diagnostic criteria, and registry methodology rather than true geographic heterogeneity. Overall, APS predominantly affects young and middle-aged women.

Thrombotic manifestations are common in APS, with venous thromboembolism representing the predominant phenotype and being strongly associated with the persistent presence of antiphospholipid antibodies (aPLA). PE may be one of the initial clinical manifestations and occurs in up to 20% of patients during the disease course, whereas arterial thromboembolic events occur less frequently and most commonly involve the cerebral circulation [16,17,18].

Mortality among patients with APS remains significantly higher than in the general population, exceeding it by approximately 50–80%, largely due to thrombotic and cardiovascular complications [17]. Myocardial infarction is relatively uncommon in APS, reported in 1–5.6% of patients [19].

Secondary APS most frequently develops in association with systemic connective tissue diseases, particularly systemic lupus erythematosus (SLE) [20]. In this population, chronic systemic inflammation and immune-mediated endothelial dysfunction further amplify thrombotic risk. Patients with SLE have a substantially increased incidence of VTE and PE compared with the general population, with PE occurring up to three times more frequently and contributing significantly to cardiovascular morbidity and mortality [21,22,23,24].

### 3.2. Epidemiology of PE-APS

PE represents the most common pulmonary manifestation of APS and occurs in approximately 14–15% of patients, as demonstrated in large European cohorts [24]. Importantly, PE constitutes one of the leading causes of cardiovascular mortality in APS, underscoring its clinical relevance [25].

Long-term observational data indicate that thrombotic events accumulate over time, with the highest incidence observed within the first years following diagnosis, although the risk persists throughout the disease course [26]. A subset of patients develops chronic thromboembolic disease (CTED) after acute PE, which may progress to pulmonary hypertension in approximately 3–4% of patients with primary APS [27].

PE may occur across all clinical forms of APS, including catastrophic antiphospholipid syndrome (CAPS), a rare but life-threatening clinical variant of APS characterized by rapid multiorgan microvascular thrombosis and high mortality [28]. Although CAPS accounts for only a small proportion of APS cases, PE appears to be more frequent in this subgroup than in classical APS [29].

PE is a common condition in the general population; however, its clinical presentation differs in patients with APS. Compared with patients with unprovoked PE without aPLA, those with APS-associated PE tend to be significantly younger. Younger age at presentation, hemoptysis, prolonged activated partial thromboplastin time (APTT), and lower Pulmonary Embolism Severity Index (PESI) scores may help identify patients in whom APS should be suspected during PE evaluation [30].

## 4. Pathologic Mechanisms in PE-APS

APS-associated PE results from a complex immune-mediated prothrombotic state driven by persistent antiphospholipid antibodies (aPLA), including lupus anticoagulant (LA), anticardiolipin antibodies (aCL), and antibodies directed against β2-glycoprotein I (anti-β2GPI) [25,31]. Among classical aPLA profiles, LA and IgA anti-β2GPI antibodies have been identified as independent risk factors for PE, whereas arterial thrombotic events such as stroke and myocardial infarction show broader associations with multiple aPLA profiles [2]. These associations are primarily derived from observational and retrospective studies and should therefore be interpreted with appropriate caution.

A particularly increased thrombotic risk has been observed in patients with so-called “triple positivity defined by the concurrent presence of LA, aCL, and anti-β2GPI antibodies. Long-term observational cohorts suggest cumulative thromboembolic event rates of up to 44% at 10 years; however, these estimates originate mainly from selected high-risk populations [10]. This immunological profile is associated with both venous and arterial thrombosis.

The relatively young age at PE onset in APS compared with the general population supports a disease-specific pathophysiology distinct from age-related thrombosis. Experimental and clinical studies indicate that aPLA induces sustained activation of endothelial cells, monocytes, platelets, and neutrophils, leading to endothelial dysfunction and amplification of inflammatory and procoagulant signaling pathways [30,32]. In particular, activation of neutrophils and the formation of neutrophil extracellular traps (NETs) have been implicated in immunothrombosis in APS; however, their prognostic value in PE-APS is currently supported mainly by indirect evidence and small cohort studies.

β2-glycoprotein I (β2GPI), rather than anti-β2GPI antibodies themselves, represents the principal autoantigen in APS. Binding of anti-β2GPI antibodies to β2GPI expressed on endothelial cells and platelets promotes procoagulant activity through multiple mechanisms, including increased tissue factor expression, impaired protein C-dependent anticoagulant pathways, and reduced fibrinolytic capacity [5]. These processes favor enhanced thrombin generation via increased activation of prothrombin, rather than increasing the synthesis of thrombin itself.

In selected PE populations, transient aPLA positivity may be detected during acute thrombotic or inflammatory states; however, only persistent antibody positivity fulfills the diagnostic criteria for APS. Observational data suggest that a small but clinically relevant subset of patients initially treated for PE—particularly those receiving direct oral anticoagulants—may ultimately meet criteria for APS upon repeated testing, underscoring the importance of reassessment in selected clinical scenarios [33].

## 5. The Main Triggering Factor—B2GPI

**Figure 1:**
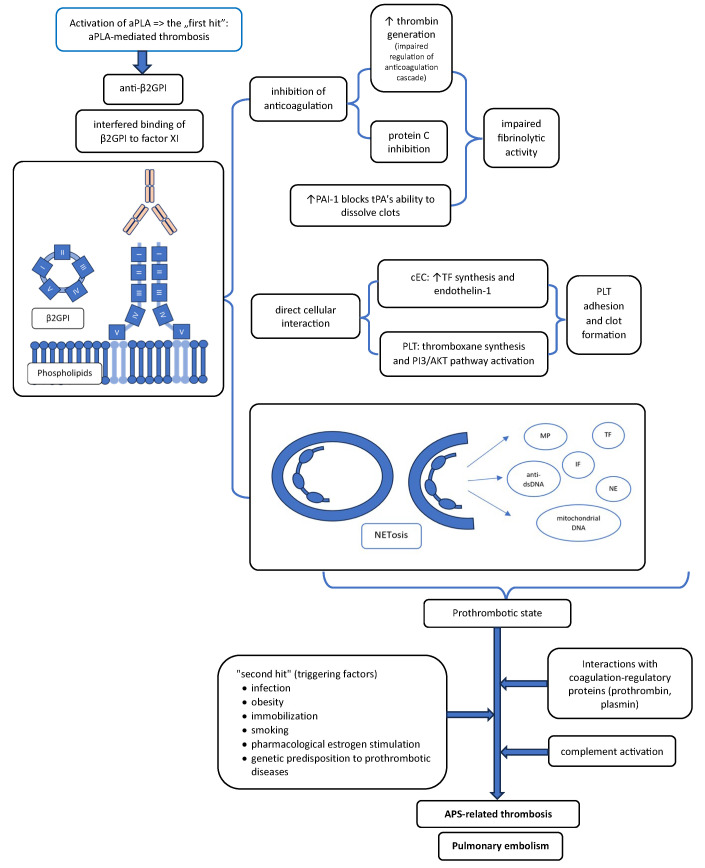
Prothrombotic activity of antiphospholipid antibodies (aPLA), particularly anti-β2-glycoprotein I antibodies. aPLA stimulates procoagulant pathways, inhibits endogenous anticoagulant mechanisms, impairs fibrinolytic activity, activates complement, and exerts direct cellular effects on endothelial cells and platelets. These processes promote a prothrombotic state and, in the presence of additional triggering factors (“second hit”), contribute to APS-related thrombosis, ultimately leading to pulmonary embolism. aPLA—antiphospholipid antibodies; β2GPI—β2-glycoprotein I; PAI-1—plasminogen activator inhibitor type 1; tPA—tissue plasminogen activator; TF—tissue factor; ECs—endothelial cells; MPO—myeloperoxidase; IFN—interferon; anti-dsDNA—anti-double-stranded DNA antibodies; NE—neutrophil elastase. The upward arrow indicates an increase in the laboratory parameter.

The “two-hit” concept in APS proposes that persistent antiphospholipid antibodies (aPLA) create a chronic prothrombotic milieu (first hit), while an additional trigger—such as infection, surgery, pregnancy, inflammation, or withdrawal of anticoagulation—is required to precipitate a clinical thrombotic event (second hit) [34,35] PE, as a manifestation of venous thromboembolism (VTE), often develops in this context [35]. The key prothrombotic mechanisms driven by aPLA, including endothelial dysfunction, platelet activation, impaired fibrinolysis, and complement activation, are schematically summarized in Figure 1.

aPLA β2-glycoprotein I (β2GPI) represents the principal autoantigen in APS. Structural alterations of β2GPI expose immunogenic epitopes, promoting the production of anti-β2GPI antibodies [3,4,5]. The binding of these antibodies to β2GPI expressed on endothelial cells and platelets induces a procoagulant phenotype through increased tissue factor expression, impairment of protein C-dependent anticoagulant pathways, and suppression of fibrinolysis [10]. In observational studies, IgA anti-β2GPI antibodies have been associated with an increased risk of thrombotic complications, particularly arterial events; however, the strength of this association varies across cohorts [36].

Anti-β2GPI antibodies further contribute to thrombosis by increasing thrombin generation through enhanced prothrombin activation and promoting platelet activation. They also disrupt the fibrinolytic balance by modulating the activity of plasminogen activator inhibitor-1 (PAI-1) and tissue plasminogen activator (tPA) [37,38,39,40,41,42,43]. These mechanisms collectively promote clot formation and maintenance rather than its physiological resolution. aPL antibodies can directly interact with endothelial cells (ECs) and monocytes through specific receptors. aPL stimulates the expression of tissue factor (TF) and endothelin-1 in endothelial cells (ECs) and monocytes [41,44]. β2GPI can bind GPIbα, a subunit of the platelet adhesion molecule. This enables the binding of anti-β2GPI antibodies, thereby influencing platelet activation. Increased thromboxane synthesis and activation of the phosphoinositide 3-kinase (PI3K)/Akt pathway lead to platelet adhesion and aggregation [42].

Inflammation-related activation of neutrophils plays an important role in APS-associated immunothrombosis. Anti-β2GPI antibodies promote neutrophil activation and the release of neutrophil extracellular traps (NETs), which amplify coagulation by activating the complement system, increasing tissue factor expression, and inactivating endogenous anticoagulant pathways [45,46]. NETs also enhance platelet activation and erythrocyte adhesion, creating a positive feedback loop that sustains thrombosis. Although increased NET formation has been associated with worse outcomes and early mortality in acute PE, these observations are largely derived from retrospective studies and small cohorts [47].

PE may occur as an isolated event but more commonly develops as a complication of deep vein thrombosis [48]. Incomplete thrombus resolution and persistent obstruction of the pulmonary arterial bed may lead to chronic thromboembolic pulmonary hypertension (CTEPH), a rare but serious long-term complication. Retrospective data suggest that CTEPH occurs more frequently in patients with APS than in those without APS; however, these findings should be interpreted cautiously due to limitations in the study design [49,50].

## 6. Clinical Presentation of Pulmonary Embolism in Antiphospholipid Syndrome

PE in the setting of APS remains one of the most serious and clinically burdensome complications, due both to the risk of acute cardiopulmonary failure and to long-term sequelae such as CTEPH. The clinical picture in APS largely mirrors that of PE in the general population, though certain features are more frequent. The most common symptoms are sudden-onset dyspnea—at rest or exertional—and pleuritic chest pain that may worsen with deep inspiration or coughing [25,51]. These symptoms are often accompanied by tachycardia, palpitations, and hypoxemia, manifesting as cyanosis, tachypnea, and fatigue. In high-risk presentations—hypotension, cardiogenic shock, and acute right ventricular (RV) failure may occur, posing an immediate threat to life [25,51]. Compared with patients without APS, those with APS more often have a history of deep-vein thrombosis (DVT) or other venous thromboembolism (VTE), and the index PE may be idiopathic, without typical provoking factors such as immobilization, recent surgery, or malignancy [52,53]. In a subset, coexisting pulmonary microthrombosis leads to a more insidious course with progressive dyspnea, declining exercise tolerance, and chronic fatigue, which can obscure early diagnosis [7]. Subclinical or mild, intermittent symptoms are also more frequent in APS and may be missed by routine assessment; episodic dyspnea, blood pressure lability, palpitations, and signs of chronic RV pressure overload—peripheral edema and reduced exercise tolerance—can be observed [48,51,54]. After an acute PE, patients with APS may develop progressive exertional dyspnea, easy fatigability, and occasionally cough or hemoptysis when microthrombosis coexists [54,55]. Epidemiologic data indicate that CTEPH develops more frequently after PE in APS than after PE without APS [53,56,57]. In the study by Zhu et al., as many as 36% of PE-APS patients developed CTEPH, and a positive LA was an independent predictor of this complication [53]. Because recurrence risk is heightened—particularly in triple-positive patients (LA + anticardiolipin [aCL] + anti-β2-glycoprotein I [aβ2GPI])—symptoms may recur or intensify, resulting in cumulative cardiopulmonary injury. Accordingly, routine surveillance of RV function, exercise testing, and early detection of CTEPH features are recommended even after a first PE episode [27,55,58].

Diagnostic pathways in APS follow standard PE algorithms but require attention to several syndrome-specific nuances [22,31]. The starting point is the assessment of clinical probability using the Wells score, the revised Geneva score, or—in hospitalized and unstable patients—the simplified Pulmonary Embolism Severity Index (sPESI) for early risk stratification [58]. The Wells and revised Geneva scores classify clinical probability (low, intermediate, high) and guide the next step, which is either D-dimer testing or direct referral for computed tomographic pulmonary angiography (CTPA). The sPESI is a prognostic tool (age > 80 years, active cancer, chronic cardiopulmonary disease, heart rate ≥ 110/min, systolic blood pressure < 100 mmHg, oxygen saturation < 90%); a score of 0 identifies low-risk patients who may be considered for outpatient management, whereas ≥1 indicates the need for in-hospital care and closer monitoring [58]. In APS, score interpretation warrants particular caution: PE may occur in the absence of classic triggers, and microthrombotic disease can yield atypical or pauci-symptomatic presentations.

For patients with a low or intermediate clinical probability, the next step is D-dimer testing. In APS, D-dimer may be chronically, modestly elevated due to microthrombosis and activation of the coagulation system, thereby reducing test specificity [48,57,59]. Elevated values mandate imaging confirmation, whereas a normal D-dimer (below 500 ng/mL or the age-adjusted threshold) safely excludes PE in low-probability patients.

CT Pulmonary Angiography (CTPA) is the current gold standard for PE diagnosis, enabling simultaneous visualization of endoluminal thrombus and quantification of RV pressure load [25]. CTPA provides high sensitivity and specificity, and multiplanar reconstructions enable a detailed assessment of both central and peripheral pulmonary arteries [26,32]. Prognostically, the RV/LV diameter ratio (≥1.0 indicates RV pressure overload) correlates with pulmonary arterial pressure and the risk of mortality [25]. Tomographic assessment may also show septal flattening, enlargement of the main pulmonary artery and inferior vena cava, and indirect signs of pulmonary hypertension [25,31]. In hemodynamically unstable patients in whom CTPA is not feasible or would delay therapy, transthoracic echocardiography (TTE) is the initial test of choice [25,31]. Echocardiographic markers of RV pressure overload include RV dilatation (RV/LV > 1), septal flattening in short-axis (“D-shape”), hypokinesia of the RV free wall with preserved apical contractility (McConnell sign), enlargement of the main pulmonary artery and inferior vena cava with reduced inspiratory collapse, and tricuspid regurgitation with elevated RV systolic pressure (RVSP > 40 mmHg) [25,31]. In technically difficult windows—e.g., obesity, mechanical ventilation, postoperative states—transesophageal echocardiography (TEE) offers superior spatial resolution and may directly visualize thrombus within the pulmonary trunk or main branches; it also evaluates left-ventricular function, interatrial shunts (PFO), and potential paradoxical embolism [25,31]. In emergencies, TEE may be the only feasible diagnostic modality. In cases of limited access to imaging, where clinical probability of circulatory instability is high, ESC guidelines allow immediate reperfusion therapy (e.g., systemic thrombolysis) before CTPA confirmation, provided there are no contraindications [25,31]. Ventilation-perfusion (V/Q) scintigraphy is preferred in selected contexts—outpatients with low clinical probability and a normal chest radiograph, young patients (especially women), pregnancy, prior contrast anaphylaxis, or severe renal impairment—while compression ultrasonography of the leg veins provides supportive evidence of PE by demonstrating DVT [25]. In APS, coagulation assays require careful interpretation; the presence of LA may artifactually prolong aPTT and complicate heparin or warfarin monitoring. Measurement of anti-Xa activity or chromogenic factor X is therefore recommended [57]. In Table 1, we present a comparison of the clinical and laboratory features of PE in patients with APS versus those without APS.

## 7. Risk Factors for Thromboembolic Complications in APS

Thromboembolic risk in APS results from the interaction between the immunological profile and coexisting clinical and environmental factors. The principal determinant is the persistent presence of aPLA at medium or high titers, confirmed after ≥12 weeks, which confers a chronic prothrombotic state. The risk is highest in patients with triple-positive serology (LA, aCL, anti-β2GPI), while isolated low-titer single positivity is associated with a lower but non-negligible thrombotic risk [32,60,61,62]. In addition to immunological factors, classical risk factors for venous and arterial thrombosis substantially modify the clinical expression of APS. A history of prior thromboembolic events, including deep vein thrombosis or PE, is the strongest clinical predictor of recurrence [59,63]. Inherited thrombophilias—such as the factor V Leiden mutation, prothrombin G20210A mutation, and protein C or S deficiency—may further increase the risk and should be considered in selected patients, particularly when planning secondary prevention strategies [30]. Hormonal and reproductive factors, including pregnancy, menopause, hormone replacement therapy, and combined oral contraceptive use, lower the thrombotic threshold in APS [60,64]. Cardiometabolic comorbidities, including obesity, dyslipidemia, hypertension, and diabetes mellitus, promote endothelial dysfunction, low-grade inflammation, and platelet activation, thereby increasing both venous and arterial thrombotic events [51,64]. Additional situational triggers include prolonged immobilization and surgical procedures, particularly orthopedic and gynecologic or obstetric interventions, which require appropriate pharmacological or mechanical thromboprophylaxis [65]. Smoking and chronic kidney disease represent further independent risk modifiers and should be actively addressed as part of long-term risk reduction strategies [51,64].

Although multiple meta-analyses confirm a higher risk of venous thromboembolism recurrence among aPL-positive patients, substantial between-study heterogeneity—related to differences in APS definitions, study populations, and follow-up duration—limits precise risk quantification [66,67,68]. From a clinical perspective, a pragmatic three-pillar model is commonly applied, integrating (i) the aPLA profile (titer, breadth, and persistence), (ii) prior thrombotic history, and (iii) coexisting clinical and environmental risk factors. This integrated approach supports individualized risk stratification and informs decisions regarding the duration and intensity of anticoagulation, including consideration of long-term or lifelong therapy in selected high-risk patients [60,69]. Regular reassessment is recommended, as both immunologic status and environmental exposures may evolve and necessitate therapeutic adjustments [65].

**Figure 2:**
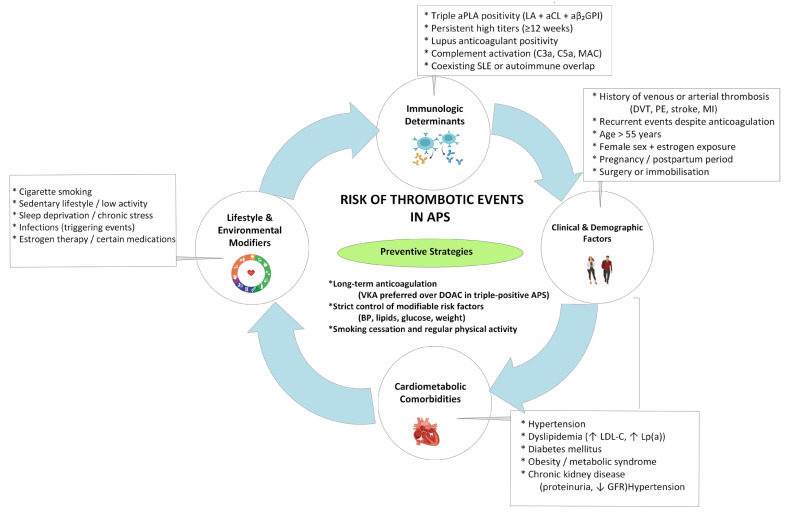
Thromboembolic Risk in Antiphospholipid Syndrome. APS—antiphospholipid syndrome; aPLA—antiphospholipid antibodies; LA—lupus anticoagulant; aCL—anticardiolipin antibodies; aβ_2_GPI—anti-β_2_-glycoprotein I antibodies; NETs—neutrophil extracellular traps; CTEPH—chronic thromboembolic pulmonary hypertension. * indicates listed clinical features. Upward arrow indicates an increase in the respective laboratory parameter and downward arrow indicates a decrease.

Routine laboratory monitoring is not required during treatment with direct oral anticoagulants (DOACs); however, when assessment of drug levels is clinically indicated, interpretation of specific assays may be challenging in the presence of aPLA, particularly LA. Comprehensive evaluation of risk factors, combined with tailored anticoagulation and lifestyle modification, remains essential to reduce thromboembolic morbidity in APS. The multifactorial nature of thrombotic risk in APS is summarized in Figure 2.

## 8. Treatment of Pulmonary Embolism in APS—Current Recommendations and Reperfusion Options

Management of PE in APS follows general PE principles but must incorporate APS-specific immunopathology and pharmacology. In the acute phase, prompt anticoagulation is paramount. In hemodynamically unstable patients or those at high risk of bleeding, unfractionated heparin (UFH) is preferred due to its rapid reversibility; otherwise, low-molecular-weight heparin (LMWH) is initiated, with subsequent transition to a vitamin Kantagonist (VKA), most commonly warfarin [25,55,59]. According to EULAR and ASH guidance, a target INR of 2.0–3.0 is recommended after a first venous event; in high-risk scenarios (e.g., recurrence at therapeutic INR, arterial events, triple positivity), some experts consider intensified anticoagulation (INR 3.0–4.0) or the addition of low-dose aspirin, balancing bleeding risk [51,55].

Data on DOACs in APS remains cautionary. The randomized TRAPS trial showed higher recurrence with rivaroxaban versus warfarin in high-risk APS, leading to strong recommendations against DOACs in triple-positive patients [56,66]. More recent observational data (2024–2025) suggest that DOACs may be acceptable only in low-risk, isolated seropositivity, but evidence remains limited [61,62]. Given monitoring challenges (LA may affect phospholipid-dependent assays), anti-Xa activity or chromogenic factor X assays are useful for reliable anticoagulation assessment [59].

In high-risk PE with shock or hypotension, systemic thrombolysis (alteplase) improves survival by rapid RV offloading [25,53]. Routine thrombolysis is not recommended for intermediate- or high-risk PE; close observation and rescue thrombolysis if the patient’s condition deteriorates are preferred [25]. Data on APS are limited, and there are no immunologic contraindications per se. Decisions should be made on an individual basis, with a careful assessment of the bleeding risk, particularly considering the potential for thrombocytopenia in APS [29]. Over the past decade, catheter-directed therapies (CDTs), including catheter-directed local thrombolysis and percutaneous mechanical thrombectomy, have emerged as alternative treatment options. ESC/EAPCI consensus details ultrasound-assisted thrombolysis and purely mechanical aspiration systems (e.g., FlowTriever or Penumbra Indigo Lightning/Flash), with trials/registries (ULTIMA, SEATTLE II, FLARE, FLASH, STORM-PE) showing rapid reversal of RV strain and low bleeding rates [49,61,63]. In APS, CDT is a reasonable option when thrombolysis is contraindicated or ineffective, provided procedures are performed in experienced centers [49,62]. In the absence of hemodynamic improvement or when thrombolysis is contraindicated, surgical pulmonary embolectomy is considered; contemporary series report single-digit postoperative mortality in specialized centers, particularly if surgery is preceded by cardiac arrest [65,69,70]. Early cardiothoracic consultation within a multidisciplinary PE response team is therefore advisable in APS [52].

**Figure 3:**
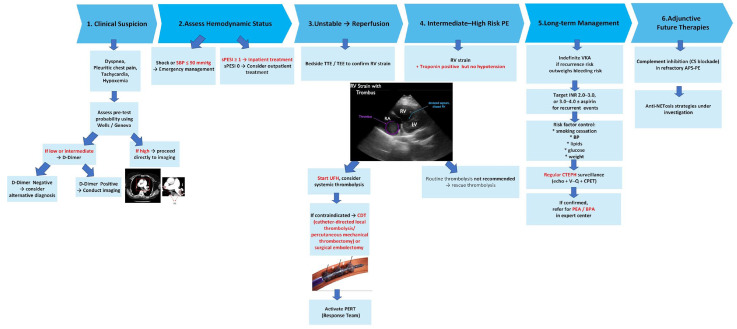
Treatment Algorithm for APS-related Pulmonary Embolism (PE). APS—antiphospholipid syndrome; LMWH—low-molecular-weight heparin; UFH—unfractionated heparin; VKA—vitamin K antagonist; DOAC—direct oral anticoagulant; CDT—catheter-directed therapy; PERT—pulmonary embolism response team; CTEPH—chronic thromboembolic pulmonary hypertension; PEA—pulmonary endarterectomy; BPA—balloon pulmonary angioplasty. Red font indicates a key clinical criterion. Plus sign (+) indicates the presence of the specified clinical feature.

After stabilization, long-term secondary prevention is instituted—typically indefinite VKA therapy when the recurrence risk outweighs the risk of bleeding [51,60]. Concomitant risk-factor modification is essential, including smoking cessation, controlling weight and blood-pressure control, treating dyslipidemia and avoiding the use of estrogens. Patients should be monitored for late complications, especially CTEPH, which is more frequent in aPLA-positive individuals; early referral to expert centers enables operability assessment and consideration of pulmonary endarterectomy (PEA) or balloon pulmonary angioplasty (BPA) [53,70]. Finally, novel targets—including complement inhibitors (e.g., C5 blockade) and anti-NETosis strategies—are under clinical investigation and may benefit PE-APS refractory to conventional therapy [69,71]. Figure 3 depicts the treatment algorithm for APS-related PE.

In summary, optimal care for PE-APS requires coordinated, multidisciplinary management and individualized decision-making. Key practical tenets include rapid anticoagulation with transition to VKA, avoidance of DOACs in high-risk APS, judicious use of thrombolysis or interventional/surgical reperfusion according to hemodynamic risk, and long-term—often lifelong—anticoagulation with active surveillance for CTEPH and other complications [25,51,52,54].

## 9. Conclusions

PE in the course of APS remains a major clinical challenge, reflecting the unique interplay between autoimmunity, endothelial dysfunction, and immunothrombosis. Although current diagnostic pathways largely mirror standard PE algorithms, APS-specific features—such as unprovoked presentations, chronically elevated D-dimer, and lupus-anticoagulant-related aPTT prolongation—require heightened clinical awareness. Long-term VKA therapy continues to be the cornerstone of management, while accumulating evidence discourages the use of DOACs in high-risk, particularly triple-positive, patients.

Where we are now: recurrence rates remain substantial despite appropriate anticoagulation, and CTEPH occurs more frequently than inPE in patients without APS, underscoring the need for systematic follow-up and early referral to specialized centers.

Where we are going: Future progress will depend on refined risk stratification that integrates detailed aPLA profiling, emerging biomarkers of complement activation and NETosis, and personalized anticoagulation strategies. Advancing therapeutic research—including complement inhibitors, anti-NETosis approaches, and targeted immunomodulation—may offer benefits for refractory disease. Multidisciplinary care and standardized long-term surveillance represent key priorities to improve outcomes and reduce morbidity in APS-related PE.

## Figures and Tables

**Table 1:** Clinical and Laboratory Features of Pulmonary Embolism in Patients with APS versus Patients without APS.

Feature	PE-APS	Non-APS PE	Key Clinical Implications
Typical trigger	Often unprovoked	Usually provoked	Absence of trigger should prompt testing for aPLA
Previous DVT	50–70%	30–40%	APS strongly linked with prior DVT [25,52]
Lupus anticoagulant	Positive (60–80%)	Negative	May prolong aPTT and interfere with monitoring [51]
D-dimer	Moderately elevated	Correlates with clot burden	Chronically increased in APS [53]
Platelet count	Low–normal	Normal	Reflects autoimmune consumption [55]
CTEPH risk	Up to 20%	<5%	Requires long-term follow-up [53,59]
Recurrence rate	25–45% at 5 years	10–15%	Warrants lifelong anticoagulation [55,57]

## Data Availability

No new data were created or analyzed in this study. Data sharing is not applicable to this article.

## Abbreviations

aPLA	Antiphospholipid Antibodies
APS	Antiphospholipid Syndrome
aPTT	Activated Partial Thromboplastin Time
β2GPI/B2GPI	Beta-2 Glycoprotein
CTEPH	Chronic Thromboembolic Pulmonary Hypertension
DOACs	Direct Oral Anticoagulants
LA	Lupus Anticoagulant
NETs	Neutrophil Extracellular Traps
PE	Pulmonary Embolism
VKA	Vitamin K Antagonists
